# Research highlights of clinical oncology early 2022

**DOI:** 10.1007/s44178-022-00006-9

**Published:** 2022-07-05

**Authors:** Kaichun Wu

**Affiliations:** Xijing Hospital of Digestive Diseases, Fourth Military Medical University, Xi’an, China

**Keywords:** Cancer, Drug therapy, Immunotherapy, Antibody, Vaccine, Biomarker

## Abstract

Advance in clinical oncology is highly relied on development of cancer research and clinical trials. There has been ample evidence regarding the new therapeutic drugs, agents or techniques for the treatment of cancer. We did a broad literature search to catch the latest progress in the cancer therapy. Here is a brief review of the newly released results of clinical trials including surgical modalities, immune checkpoint blockade, chimeric antigen receptor T-cell (CAR T) therapy, antibody–drug conjugates and tumor vaccines, etc. Biomarkers or patient assessment of cancer therapy were also discussed.

## Background

Advance in clinical oncology is highly relied on development of cancer research and clinical trials. In the last a few months there have been ample evidence regarding the new therapeutic drugs, agents or techniques for the treatment of cancer. Here is a brief review of the newly released results.

### Surgery remains the cure for most of the cancers

Gastrectomy is the standard surgery for gastric cancer. But it is not clear whether laparoscopic and open distal gastrectomy produce similar outcomes among patients with locally advanced gastric cancer. An open-label, randomized clinical trial from 14 centers in China enrolled 1056 eligible patients with non-metastatic advanced gastric cancer. At 5 years, the overall survival rates were 72.6% in the laparoscopic distal gastrectomy group and 76.3% in the open distal gastrectomy group (log-rank *P* = 0.19; hazard ratio, 1.17; *P* = 0.19). This study found that laparoscopic distal gastrectomy with D2 lymphadenectomy performed by experienced surgeons in high-volume specialized institutions resulted in similar 5-year overall survival compared with open distal gastrectomy among patients with locally advanced gastric cancer [1].

Treatment for patients with recurrent ovarian cancer has been mainly based on systemic therapy. The role of secondary cytoreductive surgery is unclear. Patients with recurrent ovarian cancer were randomly assigned to undergo secondary cytoreductive surgery plus platinum-based chemotherapy. A complete resection was achieved in 75.5% of the patients in the surgery group. The median overall survival was 53.7 months in the surgery group and 46.0 months in the no-surgery group (hazard ratio for death, 0.75; *P* = 0.02). Patients with a complete resection had the most favorable outcome, with a median overall survival of 61.9 months. In women with recurrent ovarian cancer, cytoreductive surgery followed by chemotherapy resulted in longer overall survival than chemotherapy alone [2].

Thyroidectomy is effective for the treatment of thyroid cancer, yet the postoperative administration of radioiodine (iodine-131) is controversial for patients with low-risk differentiated thyroid cancer. In a prospective randomized phase 3 trial, patients with low-risk differentiated thyroid cancer who underwent thyroidectomy and received ablation with postoperative administration of radioiodine showed a similar result of functional, structural, and biologic abnormalities of thyroid gland at 3 years to the patients with thyroidectomy only. It indicated that the postoperative radioiodine ablation may not be necessary for patients with low-risk thyroid cancer [3].

### Immune checkpoint inhibitors proved effective in more types of cancers

Unprecedented advances have been made in cancer treatment with the use of immune checkpoint blockade (ICB).Antibodies targeting the checkpoint molecules cytotoxic T lymphocyte antigen 4 (CTLA4), programmed cell death 1 (PD1) and PD1 ligand 1 (PD-L1) have had early success in the clinic in several cancer types such as melanoma, lung cancer and colorectal cancers.Patients with recurrent cervical cancer have a poor prognosis. Cemiplimab, the fully human PD1-blocking antibody demonstrated in a phase 3 trial that the median overall survival was longer in the cemiplimab group than in the chemotherapy group (12.0 months vs. 8.5 months; hazard ratio for death, 0.69; *P* < 0.001). Progression-free survival was also longer in the cemiplimab group than in the chemotherapy group (hazard ratio for disease progression or death, 0.75; *P* < 0.001). In the overall population, an objective response occurred in 16.4% of the patients in the cemiplimab group, as compared with 6.3% in the chemotherapy group. Overall, grade 3 or higher adverse events occurred in 45.0% of the patients who received cemiplimab and in 53.4% of those who received chemotherapy. Survival was significantly longer with cemiplimab than with single-agent chemotherapy among patients with recurrent cervical cancer after first-line platinum-containing chemotherapy [4]. In a randomized phase 3 trial of patients with metastatic castration-resistant prostate cancer, anit-PD-L1 monoclonal antibody atezolizumab showed a longer progression-free survival in patients with high PD-L1 IC2/3, CD8 expression and established immune gene signatures [5]. Mesothelioma is a rare and fatal cancer with limited therapeutic options. In a phase 2 trial of the anti-PD-L1 antibody durvalumab plus platinum-pemetrexed chemotherapy for patients with previously untreated, unresectable pleural mesothelioma, a median survival of 20.4 months was reached comparing that of 12.1 months with historical control.The data indicated that concurrent durvalumab with platinum-based chemotherapy has promising clinical activity for the treatment of malignant pleural mesothelioma [6].

The efficacy of immunotherapy through ICB may be enhanced by concurrent administration of immune checkpoint inhibitor and chemotherapeutic agents or target therapy drugs, even combination of two checkpoint inhibitors.These agents may involve in the stimulation of anticancer immunity either by initiating the release of immunostimulatory molecules from dying cancer cells or by mediating off-target effects on immune cell populations [7]. Nivolumab in combination with chemotherapy in 970 patients with advanced esophageal squamous-cell carcinoma was effective with a longer overall survival than control (15.4 months vs. 9.1 months; hazard ratio, 0.54; *P* < 0.001). First-line treatment with nivolumab plus chemotherapy resulted in significantly longer overall survival than chemotherapy alone in patients with advanced esophageal squamous-cell carcinoma [8]. Triple-negative breast cancer (TNBC) is defined by a lack of expression of both estrogen (ER) and progesterone (PgR) receptors as well as human epidermal growth factor receptor 2 (HER2), and is associated with poor prognosis.The addition of pembrolizumab to neoadjuvant chemotherapy led to a significantly higher percentage of patients with early triple-negative breast cancer having anevent-free survival at 36 months compared with placebo (84.5% vs. 76.8%, hazard ratio for event or death, 0.63; *P* < 0.001) [9]. There is no standard therapy for advanced endometrial cancer after failure of platinum-based chemotherapy. Study of lenvatinib plus pembrolizumab for advanced endometrial cancer showed that the median progression-free survival was longer with lenvatinib plus pembrolizumab than with chemotherapy (7.2 monthsvs. 3.8 months; hazard ratio, 0.56; *P* < 0.001). The median overall survival was longer with lenvatinib plus pembrolizumab than with chemotherapy (18.3 months vs. 11.4 months; hazard ratio, 0.62; *P* < 0.001) [10]. Apart from PD-1 and PD-L1 being targets of cancer immunotherapy, lymphocyte-activation gene 3 (LAG-3) is also identified as an immune checkpoint. The combination of relatlimab, a LAG-3-blocking antibody, and nivolumab, a PD1-blocking antibody, has been used in a phase 2–3, global, double-blind, randomized trial to patients with previously untreated metastatic or unresectable melanoma.The median progression-free survival was 10.1 months with relatlimab-nivolumab as compared with 4.6 months with nivolumab (hazard ratio for progression or death, 0.75; *P* = 0.006). Progression-free survival at 12 months was 47.7% with relatlimab-nivolumab as compared with 36.0% with nivolumab.The inhibition of two immune checkpoints, LAG-3 and PD-1, provided a greater benefit with regard to progression-free survival than inhibition of PD-1 alone in patients with previously untreated metastatic or unresectable melanoma [11].

### Novel targets or strategies of clinical therapy for cancer

Blockade of the cyclin-dependent kinase 4 and 6 pathway has been shown to be effective in the treatment of hormone receptor-positive advanced breast cancer. Cyclin-dependent kinase 4 and 6 inhibitor Ribociclibin hormone receptor-positive, HER2-negative advanced breast cancer with disease progression after endocrine therapy was investigated in double-blind, randomized, phase 3 trials. Ribociclib plus letrozole showed a significant overall survival benefit as compared with placebo plus letrozole. The median overall survival was 63.9 months with ribociclib plus letrozole and 51.4 months with placebo plus letrozole (hazard ratio for death, 0.76; *P* = 0.008) [12]. Dalpiciclib, another cyclin-dependent kinase 4 and 6 inhibitor plus fulvestrant showed a prolonged progression-free survival with dalpiciclib plus fulvestrant versus placebo plus fulvestrant (15.7 months versus. 7.2 months; hazard ratio, 0.42; *P* < 0.0001) [13].

Patients with von Hippel-Lindau (VHL) disease have a high incidence of renal cell carcinoma owing to VHL gene inactivation and constitutive activation of the transcription factor hypoxia-inducible factor 2α (HIF-2α). In a phase 2, open-label, single-group trial of the HIF-2α inhibitor belzutifan, the percentage of patients with renal cell carcinoma who had acomplete or partial response was 49%. Belzutifan was associated with predominantly grade 1 and 2 adverse events and showed HIF-2α inhibitory activity in patients with renal cell carcinomas and non-renal cell carcinoma neoplasms associated with VHL disease [14].

Autologous anti-CD19 chimeric antigen receptor T-cell (CAR T) therapy has been approved for treatment of patients with B cell acute lymphoblastic leukemia (B-ALL). But the major cause of treatment failure is antigen downregulation or loss. Dual antigen targeting could potentially prevent this, and a phase 1 trial in pediatric and young adult patients with relapsed or refractory B-ALL to test autologous transduced T cells expressing both anti-CD19 and anti-CD22 CARs was conducted. At 1 month after treatment the remission rate was 86%. The 1 year overall and event-free survival rates were 60% and 32%, with a favorable safety profile [15].

Antibody–drug conjugate demonstrated great potential to treat the patients with advanced cancers. Diffuse large B-cell lymphoma (DLBCL) is typically treated with rituximab, cyclophosphamide, doxorubicin, vincristine, and prednisone (R-CHOP), with only 60% of cure. Polatuzumab vedotin is an antibody–drug conjugate targeting CD79b which is ubiquitously expressed on the surface of malignant B cells. There wasa double-blind, placebo-controlled, international phase 3 trial of polatuzumab vedotinin patients with previously untreated intermediate-risk or high-risk DLBCL. The percentage of patients surviving without progression was significantly higher in the polatuzumab vedotin group than in the R-CHOP group (76.7% vs. 70.2% at 2 years; stratified hazard ratio for progression, relapse, or death, 0.73; *P* = 0.02) [16]. Trastuzumab deruxtecan, a HER2 antibody-drug conjugate also showed durable antitumor activity in patients with previously treated HER2-mutant non-small-cell lung cancer (NSCLC).The objective response occurred in 55% of the patients with a median duration of response 9.3 months, progression-free survival 8.2 months and overall survival 17.8 months [17].

Tumor vaccine development has been advanced in the field of therapy for melanoma. Talimogenelaherparepvec (T-VEC) is a herpes simplex virus type 1-based intralesional oncolytic immunotherapy approved for the treatment of unresectable melanoma.Neoadjuvant T-VEC plus surgery versus surgery alone for resectable stage IIIB-IVM1a melanoma was conducted in a randomized, open-label, phase 2 trial. The 2-year recurrence-free survival was 29.5% in T-VEC group and 16.5% in control group (overall hazard ratio, 0.75). The 2-year overall survival was 88.9% in T-VEC group and 77.4% in control group (overall hazard ratio,0.49). The recurrence-free survival and overall survival differences between groups persisted at 3 years. In T-VEC group, 17.1% achieved a pathological complete response.Overall,a 25% reduction in the risk of disease recurrence for neoadjuvant T-VEC plus surgery versus upfront surgery for patients with resectable stage IIIB-IVM1a melanoma [18]. In a phase 1/2 study of first-in-class immune-modulatory vaccine against indoleamine 2,3-dioxygenase (IDO) and PD-L1, patients with metastatic melanoma were treated with the vaccine targeting immunosuppressive cells and tumor cells expressing IDO and/or PD-L1 (IDO/PD-L1)combined with nivolumab.An objective response rate of 80% was reached, with 43% of complete responses and a median progression-free survival of 26 months in patients with metastatic melanoma treated with the vaccine and nivolumab [19].

### Assessment and prediction is the focus of precision medicine for cancer

It is crucial and in large demand for cancer therapies to precisely stratify patients with biomarkers or tools in order to select right drugs or options, and to achieve better efficacy and less adverse effect.The development of multigene prognostic assays has led to increased precision in estimating the absolute risk of recurrence among women with breast cancer. The recurrence score based on the 21-gene breast-cancer assay was tested in 5018 patients for predicting a chemotherapy benefit in hormone-receptor-positive, HER2-negative, axillary lymph-node-positive breast cancer.Among premenopausal women with one to three positive lymph nodes and a recurrence score of 25 or lower, those who received chemoendocrine therapy had longer invasive disease-free survival and distant relapse-free survival than those who received endocrine-only therapy. The 21-gene assay indicated chemotherapy benefit in node-positive breast cancer [20].

The applicability of circulating tumor DNA (ctDNA) genotyping to inform enrollment of patients with cancer in clinical trials has not been established. In a phase 2 trial to evaluate the efficacy of pertuzumab plus trastuzumab for metastatic colorectal cancer (mCRC), with HER2 amplification prospectively confirmed by tumor tissue or ctDNA analysis, the objective response rate of 30% in 27 tissue-positive patients and 28% in 25 ctDNA-positive patients were found as compared to an objective response rate of 0% in a matched real-world reference population treated with standard-of-care salvage therapy. Decreased ctDNA fraction 3 weeks after treatment initiation associated with therapeutic response. The ctDNA genotyping can identify patients who benefit from dual-HER2 blockade as well as monitor treatment response [21].

Weighing the risks and benefits of cancer treatment in vulnerable older adults is challenging, because they are disproportionately underrepresented in randomized clinical trials that establish the standards for cancer treatment. Therefore, vulnerable older patients with advanced cancer often receive treatments that have greater risks than benefits.Evaluation of geriatric assessment and management on the toxic effects of cancer treatmentwas carried out in a cluster-randomized trial with patients aged 70 years and older with incurable solid tumors or lymphoma and at least one impaired geriatric assessment domain who were starting a new treatment regimen.A lower proportion of patients in the assessment group had grade 3–5 toxic effects compared with the usual care group (51% vs. 71%; relative risk 0.74; *p* = 0.0001). Patients in the assessment group had fewer falls over 3 months (12% vs. 21%; adjusted RR 0.58; *p* = 0.0035) and had more medications discontinued (mean adjusted difference 0.14; *p* = 0.015).A geriatric assessment intervention for older patients with advanced cancer reduced serious toxic effects from cancer treatment. Geriatric assessment with management should be integrated into the clinical care of older patients with advanced cancer and ageing-related conditions [22].

## Data Availability

All data from public domains.

## Abbreviations

B-ALL: B cell acute lymphoblastic leukemia
CAR T: Chimeric antigen receptor T-cell
ctDNA: Circulating tumor DNA
CTLA4: Cytotoxic T lymphocyte antigen 4
DLBCL: Diffuse large B-cell lymphoma
ER: Estrogen receptor
HER2: Human epidermal growth factor receptor 2
HIF-2α: Hypoxia-inducible factor 2α
ICB: Immune checkpoint blockade
IDO: Indoleamine 2,3-dioxygenase
LAG-3: Lymphocyte-activation gene 3
mCRC: Metastatic colorectal cancer
NSCLC: Non-small-cell lung cancer
PD1: Programmed cell death 1
PD-L1: PD1 ligand 1
PgR: Progesterone receptor
TNBC: Triple-negative breast cancer
VHL: Von Hippel-Lindau
